# Supplementary material for: The interactome of intact mitochondria by cross-linking mass spectrometry provides evidence for coexisting respiratory supercomplexes
Source: Mol Cell Proteomics. 2017 Dec 8;17(2):216–32. doi: 10.1074/mcp.RA117.000470 (PMC5795388; doi:10.1074/mcp.RA117.000470)
Supplement: Supplemental Data [file supp_RA117.000470_133922_0_supp_23978_fzffwf.zip › spectra_annotation/mito_spectra_annotation/422-1-12-1-17-1.pdf]

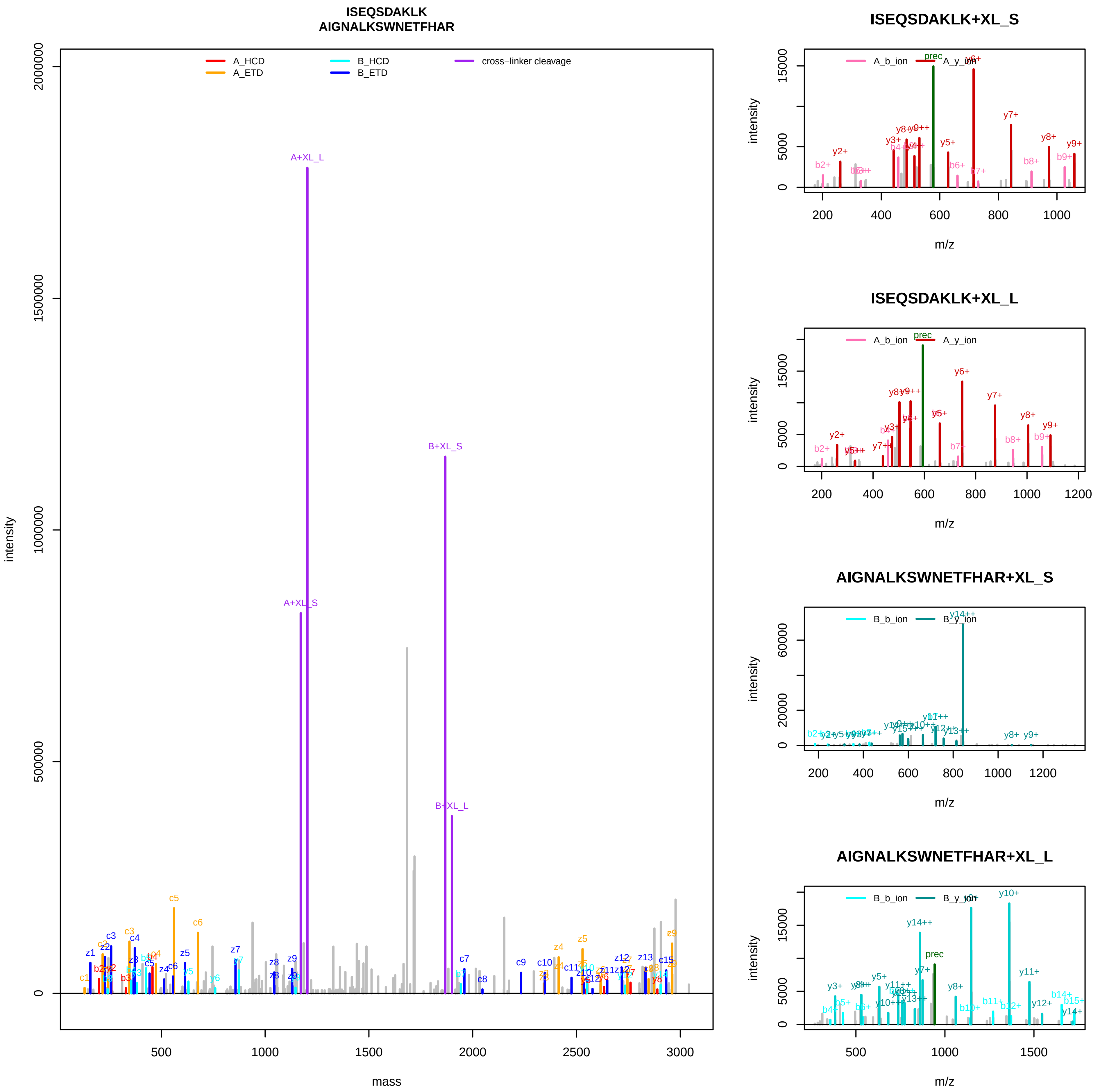

ISEQSDAKLK
AIGNALKSWNETFHAR
A_HCD
A_ETD
B_HCD
B_ETD
cross−linker cleavage
A+XL_L
A+XL_S
B+XL_S
B+XL_L
intensity
mass
0
500000
1000000
1500000
2000000
500
1000
1500
2000
2500
3000
ISEQSDAKLK+XL_S
A_b_ion
A_y_ion
prec
y6+
y7+
y8+
y9+
y5+
y2+
b2+
b6+
b7+
b8+
b9+
m/z
0
5000
15000
200
400
600
800
1000
ISEQSDAKLK+XL_L
A_b_ion
A_y_ion
prec
y6+
y7+
y8+
y9+
y5+
y2+
b2+
b7+
b8+
b9+
0
5000
15000
200
400
600
800
1000
1200
AIGNALKSWNETFHAR+XL_S
B_b_ion
B_y_ion
y14++
y11++
y8+
y9+
0
20000
60000
200
400
600
800
1000
1200
AIGNALKSWNETFHAR+XL_L
B_b_ion
B_y_ion
y10+
y14++
y11+
prec
y8+
y7+
y5+
y3+
b12+
y12+
b14+
b15+
y14+
0
5000
15000
500
1000
1500
